# Strain Engineering of Magnetic Anisotropy in the Kagome Magnet Fe_3_Sn_2_

**DOI:** 10.1021/acsnano.4c16603

**Published:** 2025-02-24

**Authors:** Deli Kong, András Kovács, Michalis Charilaou, Markus Altthaler, Lilian Prodan, Vladimir Tsurkan, Dennis Meier, Xiaodong Han, István Kézsmárki, Rafal E. Dunin-Borkowski

**Affiliations:** † Ernst Ruska-Centre for Microscopy and Spectroscopy with Electrons, Forschungszentrum Jülich, Jülich 52425, Germany; ‡ Department of Materials Science and Engineering, Southern University of Science and Technology, Shenzhen 518055, China; § HUN-REN Centre for Energy Research, Institute of Technical Physics and Materials Science, Budapest 1121, Hungary; ∥ Department of Physics, University of Louisiana at Lafayette, Lafayette, Louisiana 70504, United States; ⊥ Experimental Physics V, University of Augsburg, Augsburg 86135, Germany; # Institute of Applied Physics, Moldova State University, Chisinau 2028, Moldova; ¶ Department of Materials Science and Engineering, NTNU Norwegian University of Science and Technology, Trondheim 7491, Norway; ∇ Center for Quantum Spintronics, Department of Physics, NTNU Norwegian University of Science and Technology, Trondheim 7491, Norway

**Keywords:** strain, magnetism, anisotropy, domain wall, transmission electron microscopy

## Abstract

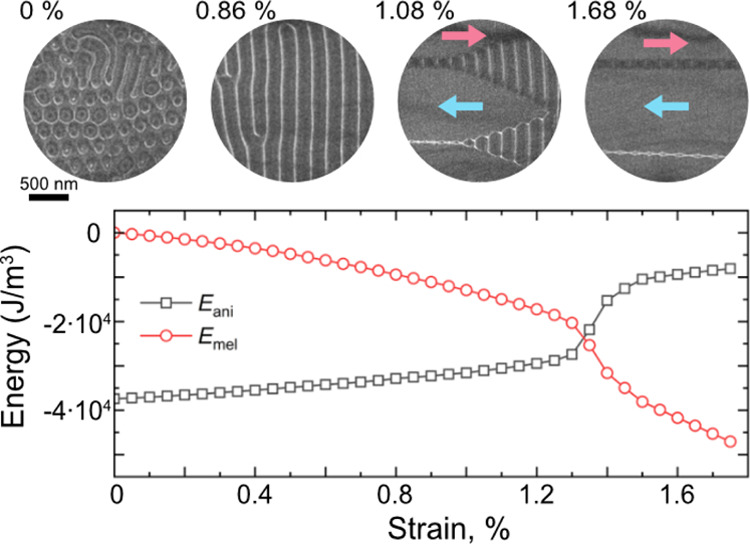

The ability to control magnetism with strain offers innovative pathways for the modulation of magnetic domain configurations and for the manipulation of magnetic states in materials on the nanoscale. Although the effect of strain on magnetic domains has been recognized since the early work of C. Kittel, detailed local observations have been elusive. Here, we use mechanical strain to achieve reversible control of magnetic textures in a kagome-type Fe_3_Sn_2_ ferromagnet without the use of an external electric current or magnetic field in situ in a transmission electron microscope at room temperature. We use Fresnel defocus imaging, off-axis electron holography and micromagnetic simulations to show that tensile strain modifies the structures of dipolar skyrmions and switches the magnetization between out-of-plane and in-plane configurations. We also present quantitative measurements of magnetic domain wall structures and their transformations as a function of strain. Our results demonstrate the fundamental importance of anisotropy effects and their interplay with magnetoelastic and magnetocrystalline energies, providing opportunities for the development of strain-controlled devices for spintronic applications.

Increasing interest in quantum materials is based in part on the synergy between strongly correlated electron states, topology and magnetism, which can lead to unconventional physical properties. Such systems include superconductors, topological semiconductors, Weyl semimetals, quantum spin liquids and two-dimensional (2D) materials, in which quantum effects are manifested over a wide range of energy and length scales.^1^ Magnetism on a kagome lattice, a 2D network of corner-sharing triangles, provides a versatile platform for investigating the interplay between magnetic phenomena, topology and electronic correlations.^2−5^ Kagome layers of 3d transition metals, stacked in perpendicular directions, offer an interesting realization of this concept. For example, kagome-type Fe_3_Sn_2_ exhibits a wide range of intriguing properties, such as flat bands near the Fermi energy,^6^ massive Dirac Fermions,^7,8^ and a large anomalous Hall effect,^7,9−11^ combined with a high Curie temperature (670 K).^12^ The material has a centrosymmetric rhombohedral structure of Fe–Sn bilayers, which alternate with Sn layers along the crystallographic *c*-axis (Figures 1a and Supporting Information Figure S1), resulting in competing uniaxial (*K*_u_) and shape anisotropies with a quality factor of <1 at room temperature. In thin films of Fe_3_Sn_2_, a competition between perpendicular magnetic anisotropy and shape anisotropy can lead to both type-I and type-II magnetic bubble formation,^13^ with diverse helicities.^14^ In such structures, the spin texture of the bubbles is nonuniform, introducing additional surface spin twists and internal degrees of freedom. Similar spin structures have also been observed in the 2D van-der-Waals-type ferromagnet Fe_5_GeTe_2_,^15^ and are typically termed unconventional bubbles^15^ or dipolar skyrmions.^14^ We use the latter name in the present work. The possibility of driving dipolar skyrmions with an electric current^16^ and of detecting them using anisotropic magnetoresistance^17^ makes Fe_3_Sn_2_ of particular interest for spintronic applications.

**Figure 1:**
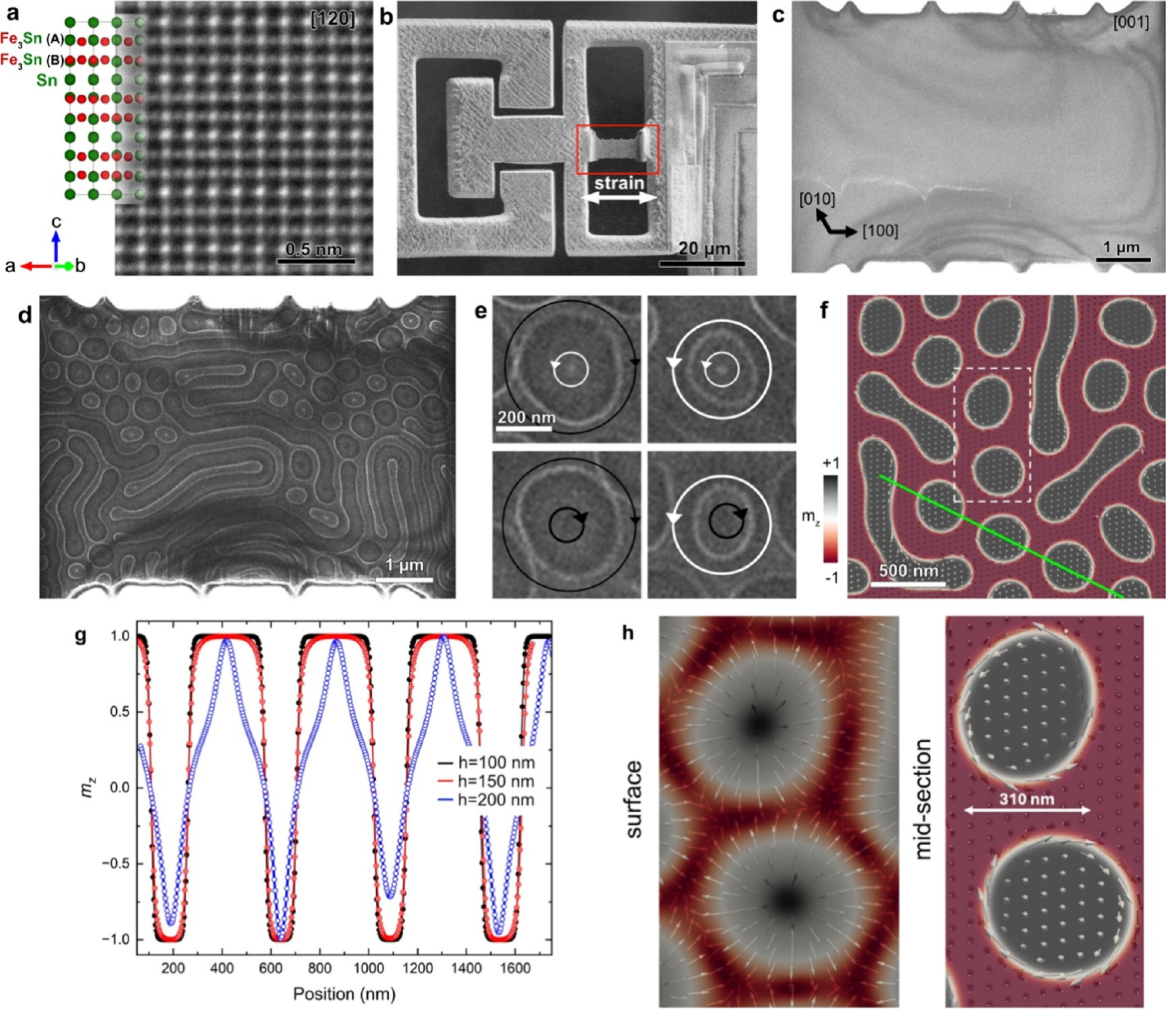
Tensile straining of Fe_3_Sn_2_. (a) Unit cell and an atomic resolution high-angle annular dark-field (HAADF) scanning TEM image of Fe_3_Sn_2_ viewed along the [120] crystallographic direction. Fe_3_Sn_2_ is composed of alternating kagome Fe_3_Sn bilayers and the hexagonal Sn layer. (b) Secondary electron SEM image of an Fe_3_Sn_2_ TEM lamella mounted on the mechanical tensile device. (c) Bright-field TEM image of the single crystal Fe_3_Sn_2_ lamella. (d) Fresnel defocus image of Fe_3_Sn_2_, showing stripe domains and dipolar skyrmions of type-I. The defocus value is 0.8 mm. (e) Selected images showing the four basic magnetic field rotation diversities of dipolar skyrmions. White and black circles indicate counterclockwise and clockwise in-plane directions, respectively. (f) Micromagnetic simulation displayed in the form of the midsection at *z* = *h*/2. (g) Line profile along the green line in (f) showing the magnetization (*m*_*z*_) at the surface and in a bulk section of the dipolar skyrmions. (h) Section of micromagnetic simulation of two dipolar skyrmions marked in (f), showing Néel-type and Bloch-type spin textures at the surface and bulk. Note the opposite rotation of the magnetization in the midsection.

In addition to applied electric currents and external magnetic fields, there is great potential in introducing and controlling strain in a material system, which adds another degree of freedom when engineering device architectures with desired properties. In spintronics, strain engineering is used to introduce magneto-elastic coupling in magnetostrictive ferromagnets.^18^ For skyrmion-hosting materials the primary driver of motion has been predominantly electric current,^19^ but topological control of skyrmions has also been achieved via strain-mediated voltage,^20^ thermally induced strain,^21,22^ and uniaxial compressive strain.^23^ Recently, Liu et al.^24^ reported strain-induced reversible motion of skyrmions using inhomogeneous uniaxial compressive strain. However, few experimental studies have been carried out to investigate local changes in strain-induced magnetic states at the nanoscale by applying a uniform tensile stress. In our previous work, we presented strain-induced-hardening in ferromagnetic Ni thin films^25^ in the presence of uniform tensile strain. Recent advances in the development of straining devices^26,27^ now allow in situ tensile straining experiments with unprecedented resolution and precision, permitting to observe and discover the coupling between mechanical deformation and nanometric magnetic states.

Here, we show how magnetic texture in Fe_3_Sn_2_, which comprises dipolar skyrmions, can be controlled by means of mechanical strain at room temperature, without the need for an electric current or external magnetic field. We select this kagome compound because it is expected to display a large strain effect as it undergoes magnetic reorientation below room temperature, driven by a change in magnetocrystalline anisotropy from easy-axis-type to easy-plane-type.^28−30^ We use Lorentz transmission electron microscopy (TEM) and off-axis electron holography to directly observe and quantify strain-induced magnetization rotation in single crystalline Fe_3_Sn_2_. We show that dipolar skyrmions merge into a periodic stripe domain structure, before the out-of-plane magnetic field gradually rotates to an in-plane direction parallel to the applied strain direction. In this state, large magnetic domains are separated by bow-tie domain walls. On removing the strain, the magnetization returns to its initial out-of-plane arrangement. The observed magnetization rotation is thought to result from the original magnetic easy-axis anisotropy being over-ridden by strain-induced anisotropy, which favors in-plane magnetic spin alignment. Such precise and reversible control of a magnetic state using mechanical strain opens new possibilities for the design of advanced spintronic devices that exploit the intricate interplay between different anisotropies.

## Results and Discussion

The Fe_3_Sn_2_ single crystal was grown by chemical transport reaction method. The chemical composition and bulk magnetic property measurements are described in the Supporting Information (Section 2). Figure 1b shows an electron-transparent Fe_3_Sn_2_ specimen mounted in a miniature straining device, which acts in the horizontal direction marked by a white arrow. The Fe_3_Sn_2_ TEM specimen is single crystalline, with no visible structural defects (Figure 1c). The viewing direction is parallel to [001] and the horizontal [100] axis is parallel to the strain direction. The energetically favorable domain state in a thin Fe_3_Sn_2_ film comprises stripe-like domains, which can be controlled by a magnetic field applied in the kagome plane. (See Figure S3 in the Supporting Information). In order to form dipolar skyrmions, a magnetic field of 0.53 T was applied parallel to the magnetic easy axis, i.e., perpendicular to the kagome plane, using the conventional objective lens of the microscope. The applied field was then removed to image the magnetic state at remanence, as shown in Figure 1d for a Fresnel defocus image of dipolar skyrmions coexisting with stripe domains. Analysis of the image contrast shows that the dipolar skyrmions have diverse helicities.^14^ Magnified images are shown in Figure 1e. The magnetic field arrangement in each dipolar skyrmion has a 2-fold rotation sense, i.e., clockwise or counterclockwise in the inner and outer regions. (See Figure S4 in the Supporting Information). A balance between ferromagnetic exchange and dipole–dipole interactions results in a hybrid Bloch-Néel spin twist, which varies through the thickness of the specimen in a three-dimensional manner.^14^ The black and white contrast in the Fresnel defocus images (Figure 1d,e) can be used to identify the dipolar skyrmions and their direction of rotation. In analogy to conventional magnetic bubbles, dipolar skyrmions in Fe_3_Sn_2_ exhibit type-I and type-II configurations. The latter configuration contains two domain walls in the peripheral region. The schematic representation of the different magnetic structures and their Fresnel defocus images can be found in Figure S5 in the Supporting Information. Figure 1f shows the result of a micromagnetic simulation of Fe_3_Sn_2_, which reproduces the observed magnetic texture. (See the Methods for details). Circular dipolar skyrmions with clockwise and counterclockwise rotations and randomly oriented stripe domains are visible. The simulation also reproduces the Bloch-type to Néel-type magnetization rotation of the dipolar skyrmions, which can be followed in the variation of the *m*_*z*_ magnetization component with depth, as shown in Figure 1g. Figure 1h illustrates the Néel-type arrangement on the surfaces and the Bloch-type arrangement in the midsections^13^ of two dipolar skyrmions extracted from the micromagnetic model.

## In Situ Tensile Straining

**Figure 2:**
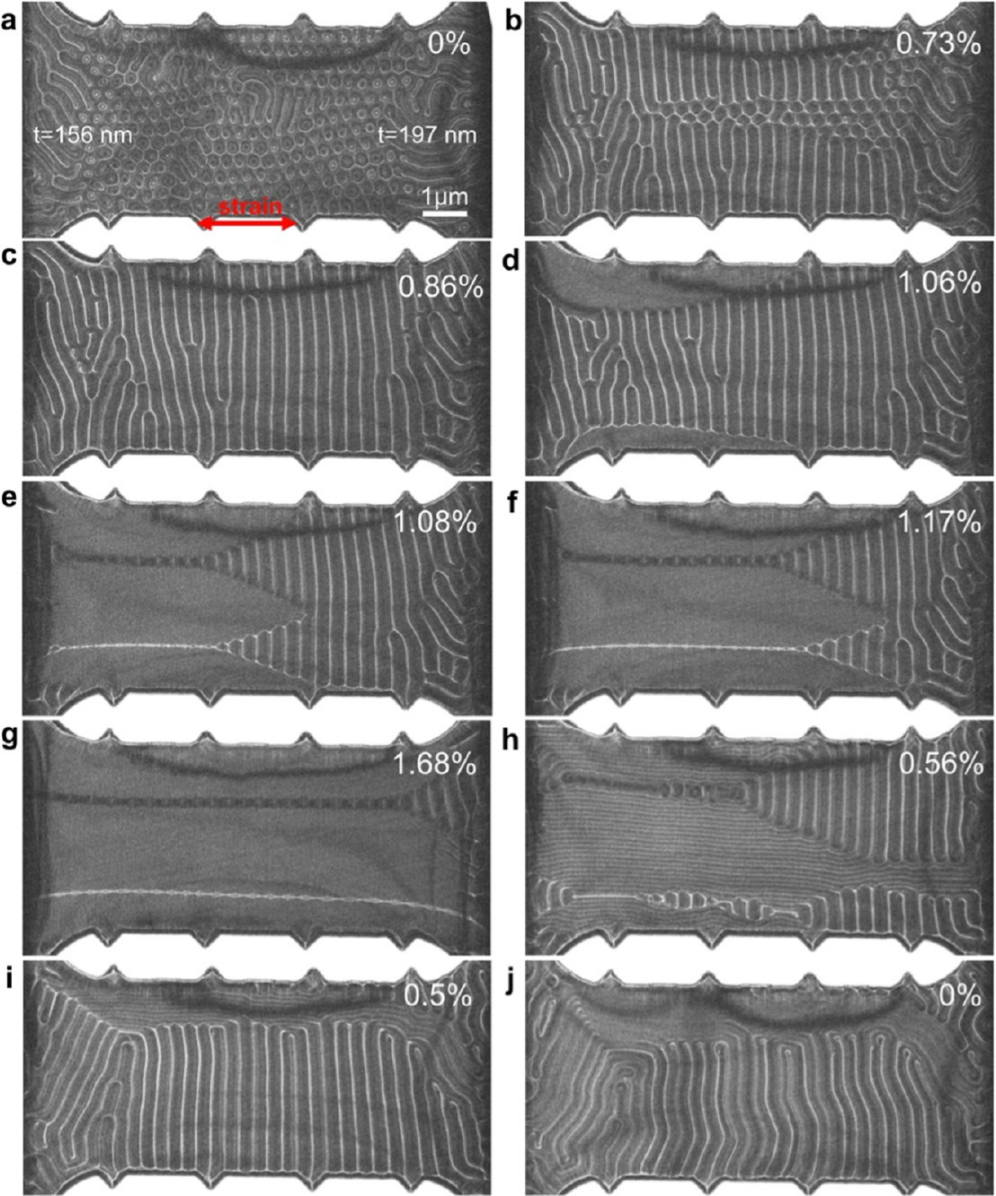
In situ tensile straining. Fresnel defocus images of (a–f) straining and (g–j) releasing of Fe_3_Sn_2_ recorded at magnetic remanence. The strain is applied along the horizontal direction, marked using an arrow in (a). The defocus value is 0.8 mm.

Figure 2 shows Fresnel defocus images of the effect of tensile strain on the magnetic state of Fe_3_Sn_2_ under magnetic field free conditions. The lateral dimensions of the specimen are approximately 10 μm × 4 μm (width × height), while its thickness varies from 156 to 197 nm within the field of view in Figure 2a. A total of 229 dipolar skyrmions was counted. In the presence of a strain of approximately 0.8%, the dipolar skyrmions were replaced by stripe domains oriented perpendicular to the strain direction (Figure 2b,c). Above a strain of 1%, uniform domains formed near the specimen edge (Figure 2d) and grew to form linear domain walls parallel to the strain direction (Figure 2e). Closure of the vertical domain walls then resulted in the formation of horizontal domain walls (Figure 2e–g). The horizontal domain walls, which were formed by vertical narrowing of the original stripe pattern, preserved the periodic contrast variation of the stripes, with a period of 190 ± 18 nm. At a maximum strain of 1.68% (Figure 2g), the specimen contained large domains separated by straight domain walls. When the strain was released (Figure 2h–j), vertical stripe domains reappeared and increased in size as the strain was reduced to zero. The transition from a vertical domain structure to large domains and back in Figures 2e–h resembles a zipper mechanism. (See Movie S1). Dipolar skyrmions were not observed in the fully relaxed state in this specimen. However, their reformation was observed in samples that were highly strained and had passed through the fracture point (see Figure S6 in the Supporting Information), suggesting that a small amount of residual strain may remain once the externally applied stress is released. The fine balance between different anisotropies in Fe_3_Sn_2_ results in a strong thickness dependence of the magnetic structure. For thicknesses below 100 nm, magnetocrystalline anisotropy is not strong enough to support dipolar skyrmions and stripe domains with out-of-plane magnetization. (See Figure S6 in the Supporting Information). Furthermore, position control of dipolar skyrmions can be realized by using mechanical strain in the presence of a magnetic field (see Movie S2), with the positions of dipolar skyrmions changing directionally in the presence of strain and returning to their original positions when strain is released.

The in situ experiment described in Figure 2 reveals three primary strain-induced processes in the Fe_3_Sn_2_ thin film: (i) the formation of a regular vertical stripe pattern by the gradual elimination and merging of dipolar skyrmions up to a strain of 1%; (ii) the formation of large domains with in-plane magnetization separated by straight domain walls (1–1.7%); (iii) recovery of the vertical stripe pattern upon strain release.

**Figure 3:**
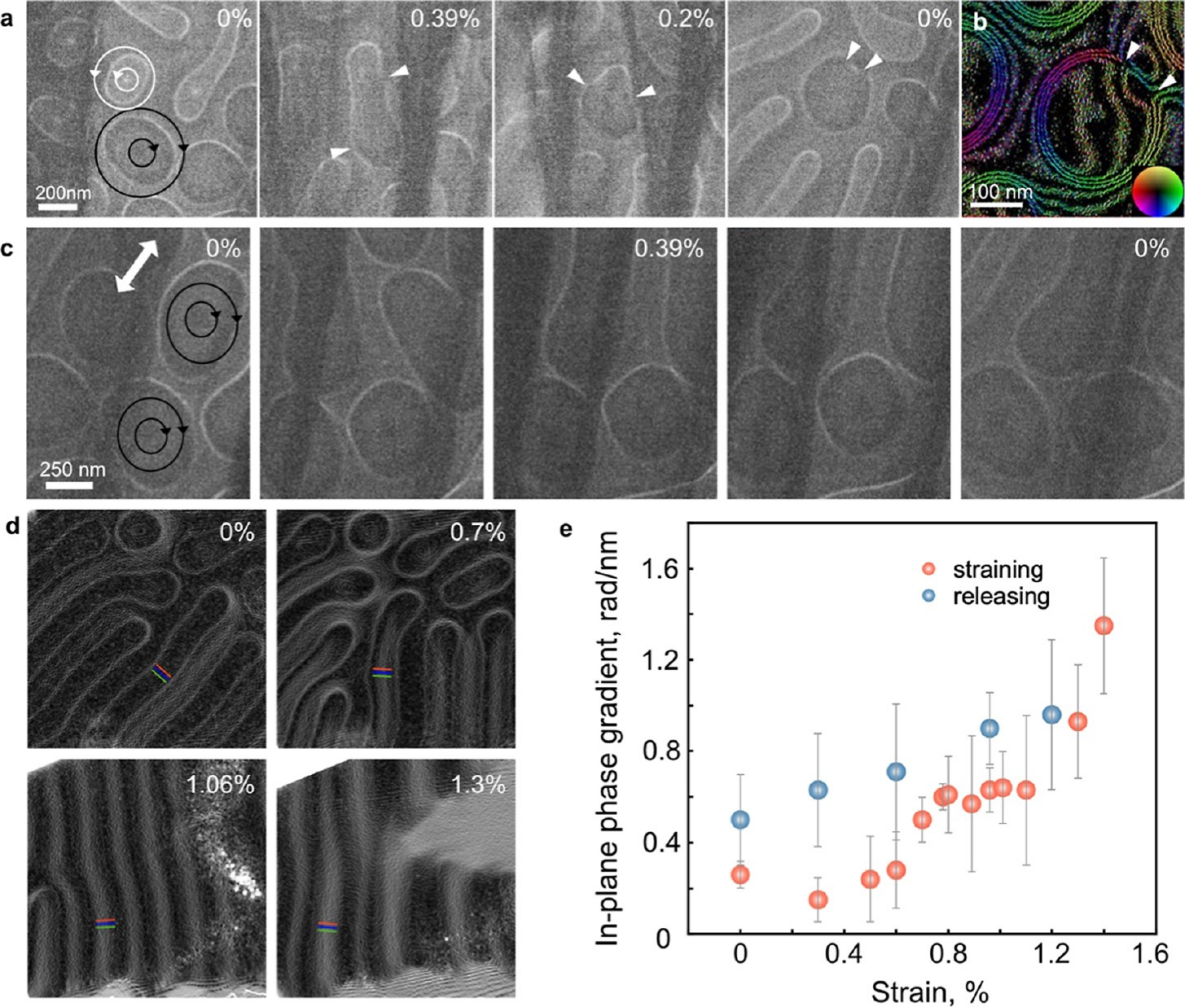
Lorentz TEM and off-axis electron holography of strain induced effects in Fe_3_Sn_2_. (a) Merging of dipolar skyrmions with opposite helicities at moderate strain. Triangular arrowheads mark domain walls. The defocus value is 0.6 mm. (b) In-plane magnetic induction map of the resulting type-II structure, measured using off-axis electron holography. The direction of the projected in-plane magnetic induction is visualized according to the color wheel. (c) The antiparallel field alignment at the peripheries of two dipolar skyrmions is robust against skyrmion merging at high strain. The defocus value is 0.6 mm. (d) Formation of stripe domains perpendicular to the strain direction. The marked region was used to estimate the in-plane phase gradient values plotted in (e), which reveal changes in projected in-plane magnetic induction as the strain increased (red circles) and then released (blue circles). The error bars correspond to the standard deviations of the phase gradient.

At moderate strains (up to 0.7%), the dipolar skyrmions merge via two characteristic mechanisms, which are presented in Figure 3a–c. In the first case, clockwise and counterclockwise rotation of the magnetization rotation of adjacent dipolar skyrmions is associated with parallel field alignment at their peripheries. In the presence of strain, the dipolar skyrmions merge to form an elliptical shape, whose opposite ends maintain the original magnetization rotation. The outer edge of the newly formed particle contains two domain walls, which form a type-II structure and are marked by arrowheads in Figure 3a. No core structure is observed in the merged particle, suggesting the presence of a hybrid texture,^14^ in which the upper and lower surfaces of the skyrmion have opposite helicities. As the strain is released, the two domain walls move closer together, until a small segment of about 50 nm remains between them. Figure 3b shows a magnetic induction map of such a type-II structure measured using off-axis electron holography. At the two domain walls, the in-plane field directions are head-to-head and tail-to-tail. A similar merging process of skyrmions with opposite helicities to form a particle with a complex spin structure is presented in Figure S7 in the Supporting Information. In the second case (Figure 3c), clockwise rotation of the magnetization of adjacent dipolar skyrmions is associated with antiparallel field alignment at their peripheries. This configuration remains robust against merging up to higher strain levels.

We analyzed the internal magnetic field alignment in the vertical stripe domains. Figure 3d shows the gradient of the electron optical phase measured using off-axis electron holography at different strain levels. As the electron optical phase is sensitive to in-plane field components projected in the incident electron beam direction, field rotation inside the Fe_3_Sn_2_ specimen can be inferred from the phase gradient measured at the same location as a function of strain. The in-plane phase gradient in Figure 3e increases significantly as the strain is increased to 1.30%, which is consistent with the magnetization in the sample rotating from out-of-plane to in-plane.

The most striking and unexpected observations of the strain-release cycle in the Fe_3_Sn_2_ thin film presented in Figure 2 are that the configuration changes to domain walls that are perpendicular to the strain axis before aligning with the strain axis, and that the initial out-of-plane magnetization direction rotates away from the original magnetocrystalline anisotropy axis and aligns in-plane in the presence of strain before returning reversibly to the out-of-plane direction when the strain is released.

## Tuning of Magnetocrystalline Anisotropy Energy via the Magnetoelastic Effect

**Figure 4:**
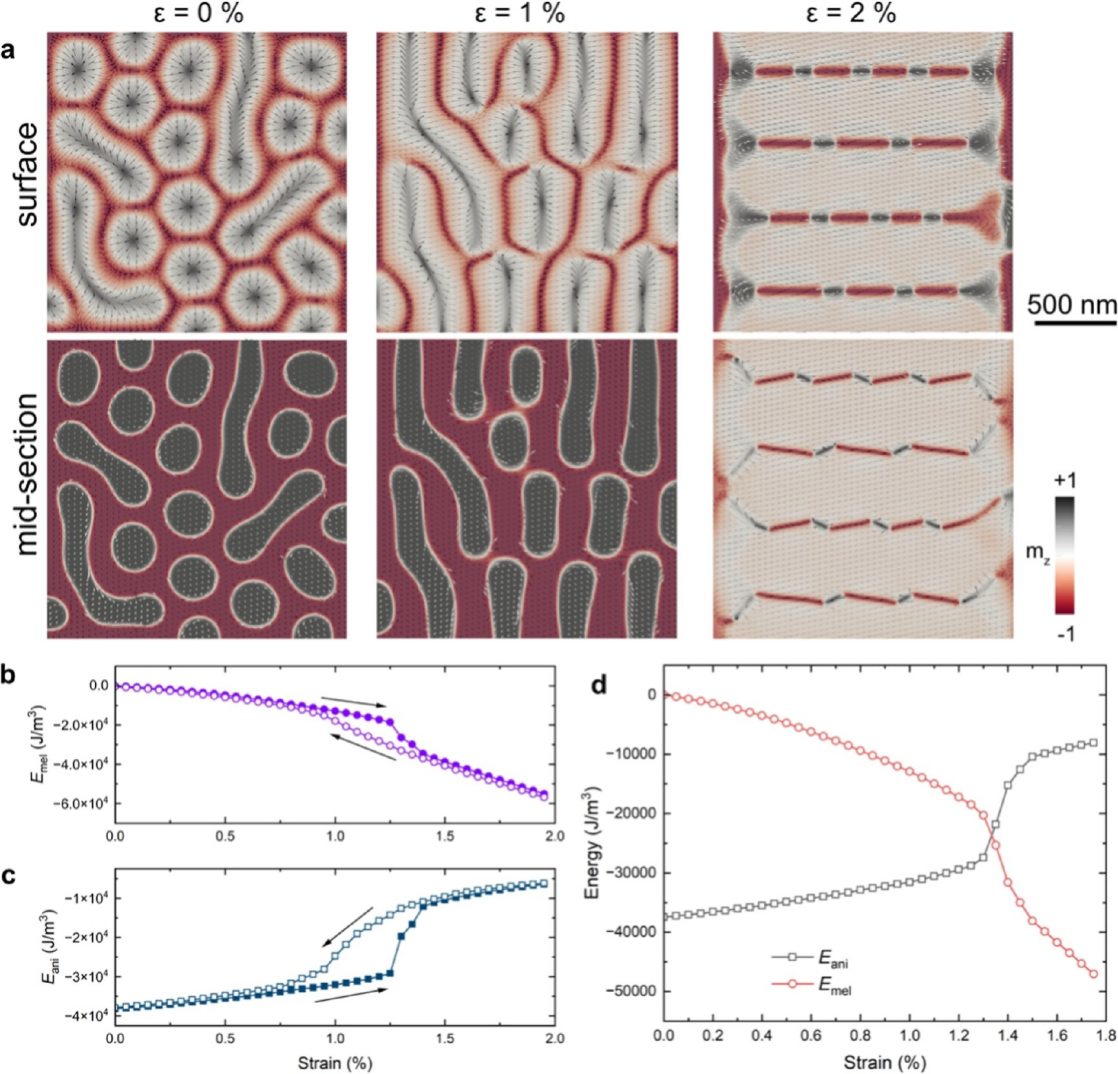
Micromagnetic simulation results. (a) Upper and middle sections, showing Néel and Bloch arrangements as a function of strain (0, 1 and 2%). (b,c) Calculated anisotropy energy (*E*_ani_) and magnetoelastic energy (*E*_mel_), revealing hysteretic behavior during strain loading and unloading. (d) The onset of in-plane magnetization occurs when the magnetoelastic energy density becomes greater than the magnetocrystalline anisotropy energy.

Figure 4 shows micromagnetic simulations of the magnetic state of the Fe_3_Sn_2_ lamella at different strains. The first row from the top shows the state of the surface of the lamella, while the second row shows the state at half of the lamella thickness (*z* = *h*/2).

In these simulations, the first-order magnetoelastic constant *B*_1_ was used as a fitting parameter to achieve a match with the experimental results. The value of *B*_1_ determines the strain at which the magnetoelastic energy *E*_mel_ becomes larger than the original magnetocrystalline uniaxial anisotropy energy *E*_ani_. By comparing the simulations with the experimental results, in which in-plane rotation of the magnetization was observed at ϵ ≈ 1.3%, a value for *B*_1_ of −3100 ± 100 J/m^3^ was obtained. Significantly, we find that the simulations reproduce all of the key experimental features: (i) the initial state that contains stripe domains and dipolar skyrmions with both helicities; (ii) merging and rotation of the stripe domains and dipolar skyrmions perpendicular to the strain axis with increasing tensile strain along the *x* axis; (iii) abrupt rotation of the magnetization to the in-plane direction at a critical strain of ϵ ≈ 1.3%; (iv) return of the system to a state comparable to the original state upon decreasing the strain, while exhibiting hysteretic behavior.

Based on the simulations and on the analysis of the domain wall structures, we propose that the hysteretic behavior results from configurational anisotropy,^31^ i.e., it is associated with the topology of the magnetization textures and the processes by which the local magnetization twists to eliminate stripe domains. The hysteretic effect seen in Figure 4b,c is in agreement with the experimental observations, as seen in Figure 2 where the transfer of the magnetization in and out of the in-plane configuration occurs at different strain levels in the loading and unloading process.

The formation of stripe domains is unexpected. Conventionally, the magnetization would be expected to gradually turn toward the strain direction and not to form domains perpendicular to it. Our analysis here reveals that the formation of stripe domains perpendicular to the strain axis satisfies exchange energy, magnetoelastic energy and magnetostatic energy, in contrast to the penalty in magnetocrystalline anisotropy energy. At a critical strain of 1.3%, there is a drastic change in the energy balance, with *E*_ani_ increasing by 50%, while at the same time *E*_mel_ decreases by 50% (see Figure 4d).

## Domain Wall Width Analysis

**Figure 5:**
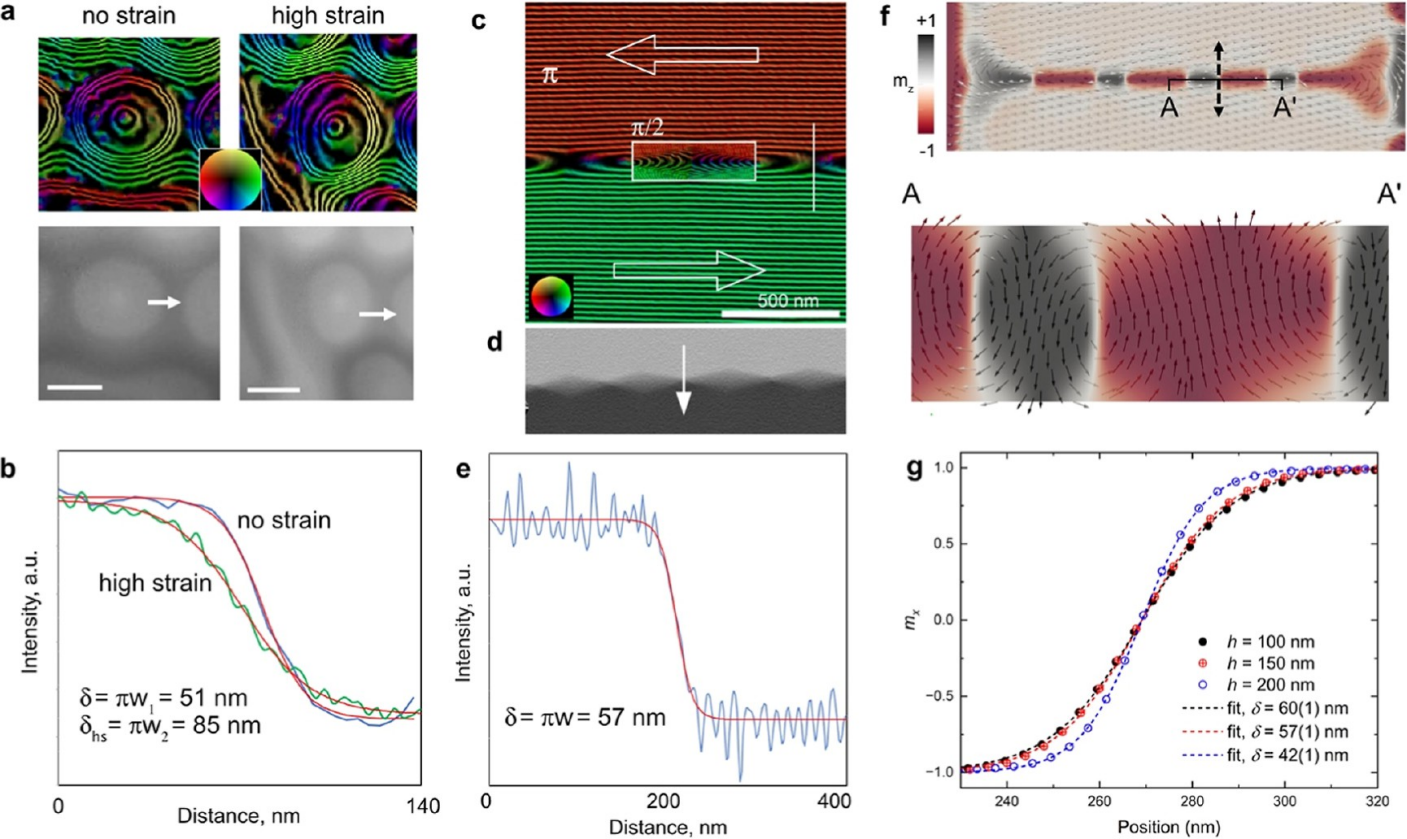
Domain wall width analyses of Fe_3_Sn_2_. (a) Magnetic induction maps and phase shift images of dipolar skyrmions imaged in relaxed (no strain) and high strain conditions. The phase contour spacing is 2π/6 radians. The scale bar is 200 nm. (b) Line profiles extracted from the phase shift images in (a) across domain walls at the positions of the arrows. The line profiles are fitted using tanh functions. The measured domain wall width increases from 51 ± 9 to 85 ± 9 nm with strain for dipolar skyrmions. (c) Magnetic induction map of in-plane magnetic domains imaged at high strain. Arrows and colors highlight the 180° alignment of the in-plane magnetic field directions. The contour spacing in the rectangular box is increased to π/2 radians to visualize the induction lines of the bow-tie domain wall. (d,e) Differential of the phase shift and line profile used to determine a value for the domain wall width of 57 ± 9 nm. (f) Micromagnetic simulation of in-plane magnetic domains with a bow-tie wall showing the surface (top). The cross section marked (A–A′) reveals the depth variation of the field direction in the bow-tie domain wall. The double-head arrow indicates the location of the linescan of *m*_*z*_ shown in (g). The fitting reveals the wall width variation.

The off-axis electron holography results and micromagnetic simulations allow the domain wall structure in thin films of Fe_3_Sn_2_ to be analyzed, in particular for the bow-tie domain wall that forms at high strain levels. Figure 5 shows experimental and theoretical analyses of domain walls in dipolar skyrmions and bow-tie domain walls. Domain wall width measurements were carried out for dipolar skyrmions in the initial and highly strained conditions (Figure 5a). Figure 5b shows phase profiles extracted across the edges of dipolar skyrmions marked by arrows in Figure 5a. Each profile was fitted using a hyperbolic tangent function, according to the expression *y = y*_0_ + *a* tanh((*x* – *x*_0_)/*w*), where *y*_0_, *a*, *x*_0_ and *w* are constants obtained from the fit. The width of the domain wall can be defined as δ *=* π*w*. In this way, the domain wall width for Fe_3_Sn_2_ dipolar skyrmions was determined to be 51 ± 9 nm for the initial condition and 85 ± 9 nm for the strained condition. Figure 5c shows a magnetic induction map of a bow-tie domain wall measured using off-axis electron holography. The parallel contour lines and colors confirm the 180° alignment of the projected in-plane field. The inset to Figure 5c shows part of the magnetic induction map with a phase contour spacing of π/2 radians, which helps to visualize the field lines at the domain wall. The decrease in contour spacing in the middle section suggests out-of-plane magnetic field alignment. The term “bow-tie domain wall” is inspired by the well-known cross-tie domain wall structure (see page 225 in Ref. (32)), which comprises 90° segments of circular and cross Bloch lines. In the case of a cross-tie domain wall, the in-plane magnetic induction is uniform, and the density of the induction lines is constant. Experimental measurements of a conventional cross-tie wall in a soft ferromagnetic alloy using off-axis electron holography were presented in ref (33) (Figure 5, p.74). In contrast, the bow-tie wall presented here contains segments where the magnetic field is oriented in the out-of-plane direction (see Figure S5 in the Supporting Information). By using the phase gradient (Figure 5d), the domain wall width was measured to be 57 ± 9 nm (Figure 5e), which is similar to the value measured for unstrained dipolar skyrmions. Figure 5f shows a top view and a cross-section of a bow-tie domain wall extracted from the micromagnetic simulation along the line A–A′. The arrows show an alternation of out-of-plane orientation and rotation within the segments.

In our micromagnetic simulations of dipolar skyrmions, we used the expression presented above to fit profiles of the *z* component of the magnetization at different positions, since the domain wall is not uniform through the thickness of the sample. In the center of the lamella (*z* = *h*/2), the width is 43 ± 2 nm, whereas between the center and the surface (*z* = 3*h*/4) it is 55 ± 2 nm, yielding an average domain wall width of 49 ± 2 nm, in close agreement with the experimental result presented in Figure 5b. In contrast, the domain wall thickness in the strained state (Figure 5f) is larger in the center of the lamella (60 ± 2 nm) and becomes smaller toward the surfaces (42 ± 2 nm), yielding an average domain wall width of 51 ± 2 nm.

## Conclusions

Our study of mechanical-strain-induced effects on magnetic states in thin films of kagome-type Fe_3_Sn_2_ using in situ magnetic imaging in the transmission electron microscope and a MEMS-based straining nanodevice reveals the elimination and merging of dipolar skyrmions, the formation of regular stripe domains and rotation of the preferred out-of-plane magnetization direction to in-plane as the strain is increased, while the system stays in the elastic regime. We identify two dipolar skyrmion merging mechanisms under low strain conditions and the formation of a bow-tie domain wall, which comprises both in-plane and out-of-plane field components, in a highly strained condition. Micromagnetic simulations reveal a hysteretic strain effect on the anisotropy and magnetoelastic energies and show that the formation of stripe domains perpendicular to the strain axis satisfies exchange, magnetoelastic and magnetostatic energies. Our observations highlight the potential of strain-controlled magnetism in nanomagnetic devices and underscore the importance of localized magnetic textures in the interplay between anisotropic effects, magnetoelastic energies and magnetocrystalline properties for advancing practical applications in technologies, including strain-mediated magnetic random-access memory (MRAM), strain-based magnetic sensors, flexible electronics, and magnetostrictive energy harvesters.

## Methods

### In Situ Mechanical Straining

The stress–strain experiments in the transmission electron microscope were performed using a Bestron (Beijing) Science and Technology Co., LTD in situ double tilt TEM specimen straining holder (INSTEMS-MET).^34^ Focused Ga ion beam sputtering in a dual beam scanning electron microscope (ThermoFisher Helios NanoLab 460F1) was used to position and thin an Fe_3_Sn_2_ single crystal that had been prepared using a chemical transport method.^13^ In the process, a slice of 9 × 6 × 2 μm^3^ in dimension was extracted from Fe_3_Sn_2_ and then loaded on the microelectromechanical systems (MEMS) chip by the piezo controlled easy-lift needle and welded by ion beam-induced deposition of Pt inside the FIB instrument. Then the specimen was thinned by the Ga ion beam with the ion beam current gradually reduced from 2.3 nA to 80 pA. Markers were fabricated at the edge of the lamella for strain measurement, as shown in Figure 1b. Strain was applied to the specimen in the Bestron holder using the MEMS chip, which contains a lead zirconate titanate (PZT) actuator for displacement control. In this setup, a hook catches a T-head to realize a uniaxial tensile test of the TEM specimen. The PZT actuator can apply load up to 1 N with a resolution of 5 nN corresponding to a stress of ∼4 GPa. The specimen can be tilted to the longitudinal and transverse axes by ±15°. Besides mechanical straining, the MEMS technology allows to apply additional heating and electrical biasing or their combination when the appropriate MEMS device is selected. Details of specimen preparation and the in situ straining set up can be found in ref (35), US Patent, No: US 10,103,001 B2.

### Magnetic Imaging

Images of magnetic domain walls and projected in-plane magnetic induction were recorded and quantified using Fresnel defocus imaging and off-axis electron holography. Off-axis electron holograms were recorded using a spherical aberration corrected TEM (ThermoFisher Titan 60–300) operated at 300 kV. Magnetic-field-free conditions were realized by switching off the conventional microscope objective lens and using the transfer lens of the aberration corrector for imaging. Fresnel defocus images and off-axis electron holograms were recorded on a 4k × 4k pixel direct electron counting detector (Gatan K2 IS). The typical biprism voltage was 90 V, which corresponded to a holographic interference fringe spacing of 3.02 nm and holographic interference fringe contrast measured in vacuum of 60%. Image analysis was performed using Gatan Microscopy Suite and HoloWorks software. Total phase shift information was extracted using a standard Fourier transform method. The total phase shift provides information about local variations in both electrostatic and magnetic potential. Since the lamella is a single crystal with negligible thickness variations, it was assumed that the electrostatic contribution to the signal is constant and that any variations in phase away from the sample edge are magnetic in origin. Magnetic induction maps were generated by in the form of contours obtained from the recorded phase images and colors determined from their gradients.

### Micromagnetic Modeling

The Fe_3_Sn_2_ sample was modeled as a ferromagnet with exchange stiffness *A*, magnetoelastic coupling *B*_1_, saturation magnetization *M*_s_ and first-order and second-order perpendicular magnetocrystalline anisotropies *K*_1_ and *K*_2_, respectively. The magnetoelastic energy density of a hexagonal lattice is^36^
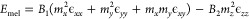

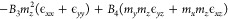


To the best of our knowledge, these constants are not known for Fe_3_Sn_2_. However, in our experiments tensile strain was only applied along the *x* axis. Therefore, the only nonvanishing terms in the above equation are the *B*_1_*m*_*x*_^2^ϵ_*xx*_ and -*B*_3_*m*_*z*_^2^ϵ_*xx*_ terms. Given that the latter term has the same *m*_*z*_ dependence as the first-order uniaxial anisotropy, we set *B*_3_ = 0 and considered only the first order magnetoelastic constant *B*_1_ in our approximation. The energy density of our model then takes the form
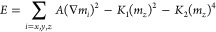

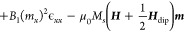
where *m*_(__*i=x,y,z)*_ are components of the magnetization unit vector ***m*** = ***M***/*M*_s_, ***H*** is the external field vector. *H*_dip_ is the magnetic field vector due to long-range dipole–dipole interactions and ϵ is the mechanical strain.

The values of the saturation magnetization and the two uniaxial anisotropy constants were measured experimentally and found to be *M*_s_ = 5.66 × 10^5^ A/m, *K*_1_ = 5.2 × 10^4^ J/m^3^ and *K*_2_ = 6.5 × 10^3^ J/m^3^, respectively. Our value for *M*_s_ is closer to that reported in ref (37) compared to that reported in refs (14 and 38). Our value of the first-order uniaxial anisotropy constant is in good agreement similar to Refs (14,38,39). For the exchange stiffness, we estimated a value by comparing the Curie temperature of Fe_3_Sn_2_ with that of pure Fe, and obtained a value of *A* = 12 × 10^–12^ J/m, which is higher than the value reported in refs (14, 38 and 39) and lower than the value estimated in ref (37) from the experimentally determined domain wall width. Our approach for estimating *A* provides a more accurate estimate because the Curie temperature can be measured more accurately than the domain wall width, which depends on the imaging resolution and sample thickness (cf.^25^).

In our sample, the exchange length is  = 7.7 nm. In the simulation, the sample had dimensions of 2000 nm × 2000 nm × 200 nm, which is comparable to the dimensions of the sample used in our experiments. The cell size was 4 nm × 4 nm × 4 nm, and the micromagnetic simulations were performed using Mumax3.^40^ Tests using other cell sizes were performed to confirm numerical stability. The simulation protocol was to start the system with an initial state of **m** = (0,0,1) and numerically integrate the Landau–Lifschitz–Gilbert (LLG) equation ∂_*t*_***m*** = −γ(***m*** × ***H***_eff_)+α(***m*** × ∂_*t*_***m***), where ***H***_eff_ = −∂*E*/∂***m***μ_o_***M***_**s**_ is the effective field. The LLG equation was integrated for 20 ns until the system reached equilibrium in zero field and for zero strain. Then, the tensile strain ϵ_*xx*_ was increased gradually from 0 to 2% in steps of 0.05%. At each step, the LLG equation was integrated numerically for 2 ns to allow the system to reach a new equilibrium.

## Supporting Information Available

Description of Fe_3_Sn_2_ crystal structure; crystal growth and characterization of Fe_3_Sn_2_, composition and magnetic properties; magnetic states observed in Fe_3_Sn_2_; helicities of dipolar skyrmions observed in phase shift images; schematics and Fresnel images of the magnetic structures in Fe_3_Sn_2_; mechanical fracture test of a TEM lamella; merging process of dipolar skyrmions of opposite helicities (PDF)
Strain-release cycle (AVI)
Control of positions of magnetic skyrmions by stress (AVI)

## Supplementary Material

nn4c16603_si_001.pdf

nn4c16603_si_002.avi

nn4c16603_si_003.avi
