# Supplementary material for: Strain Engineering of Magnetic Anisotropy in the Kagome Magnet Fe3Sn2
Source: ACS Nano. 2025 Feb 24;19(8):8142–51. doi: 10.1021/acsnano.4c16603 (PMC11887483; doi:10.1021/acsnano.4c16603)
Supplement: Supplementary file 1 — nn4c16603_si_001.pdf [file nn4c16603_si_001.pdf]

Supporting Information:

Strain engineering of magnetic anisotropy in the kagome magnet Fe_3Sn_2

Deli Kong[1,2], András Kovács[1,3*], Michalis Charilaou[4*], Markus Altthaler[5], Lilian Prodan[6], Vladimir Tsurkan[5,6], Dennis Meier[7,8], Xiaodong Han[2*], István Kézsmárki[5], Rafal E. Dunin-Borkowski[1]

[1] Ernst Ruska-Centre for Microscopy and Spectroscopy with Electrons, Forschungszentrum Jülich, Jülich, 52425, Germany.
[2] Department of Materials Science and Engineering, Southern University of Science and Technology, Shenzhen, 518055, China.
[3] Institute of Technical Physics and Materials Science, HUN-REN Centre for Energy Research, Budapest, 1121, Hungary
[4]Department of Physics, University of Louisiana at Lafayette, Lafayette 70504, USA.
[5] Experimental Physics V, University of Augsburg, Augsburg, 86135, Germany.
[6] Institute of Applied Physics, Moldova State University, Chisinau, MD 2028, Moldova.
[7] Department of Materials Science and Engineering, NTNU Norwegian University of Science and Technology, Trondheim, 7491, Norway.
[8] Center for Quantum Spintronics, Department of Physics, NTNU Norwegian University of Science and Technology, Trondheim, 7491, Norway.

*Corresponding author(s), Email(s): a.kovacs@fz-juelich.de, michalis.charilaou@louisiana.edu, hanxd@sustech.edu.cn

This file contains:

Supporting Figs S1-S7 and accompanying captions and text.

Captions for Supporting movies S1 and S2.

1. The frustrated Fe_3Sn_2 kagome magnet

Figure S1 shows the crystal structure of Fe_3Sn_2, which is best described by space group *R*-3*m*, space group number 166 and lattice parameters $a = b = 0.53147$ nm, $c = 1.97025$ nm. The Fe and Sn layers form bilayers with offset kagome networks. The kagome layers are made up of two sizes of equilateral triangles with slightly different Fe-Fe distances. The Fe site is characterized by two adjacent breathing kagome layers forming bilayer blocks (or double kagome slabs). These atomic arrangements lead to spin frustration, with non-collinear ferromagnetic and topological properties. The bilayer structure gives rise to the interlayer hybridization due to the multiple *d* orbitals of Fe atoms leading to deviations from the single-orbital 2-dimensional kagome lattice scenario (see A. Mielke, Exact ground states for the Hubbard model on the kagome lattice, J. Phys. A 25, 4335 (1992).)

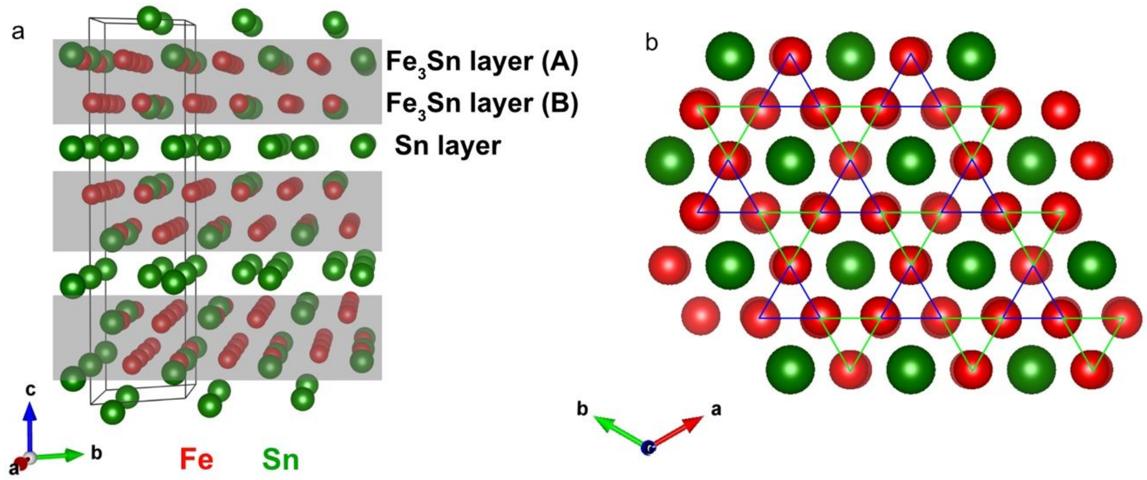

Figure S1. Atomic structure of Fe_3Sn_2 viewed along (a) the a-axis and (b) the c-axis. In (a), the frame marks the unit cell. In (b), corner-sharing equilateral triangles form a two-dimensional kagome lattice.

2. Crystal growth and characterization

High-quality single crystals of Fe_3Sn_2 were grown by the chemical transport reaction method, following a procedure similar to that described in Refs [7,13]. The previously sintered high purity polycrystalline Fe_3Sn_2 was inserted in an evacuated quartz ampule together with 10 wt.% of iodine, which served as the transport agent. The growth was performed in a two-zone horizontal furnace with a temperature gradient of about 680-740ºC. After six weeks of transport, shiny hexagonal plates were obtained in the hot part of the ampule, with lateral dimensions up to 4 mm and thicknesses of about 20-60 μm.

An optical image of the as-grown Fe_3Sn_2 crystal selected for this study is shown in the inset of Figure S2a.

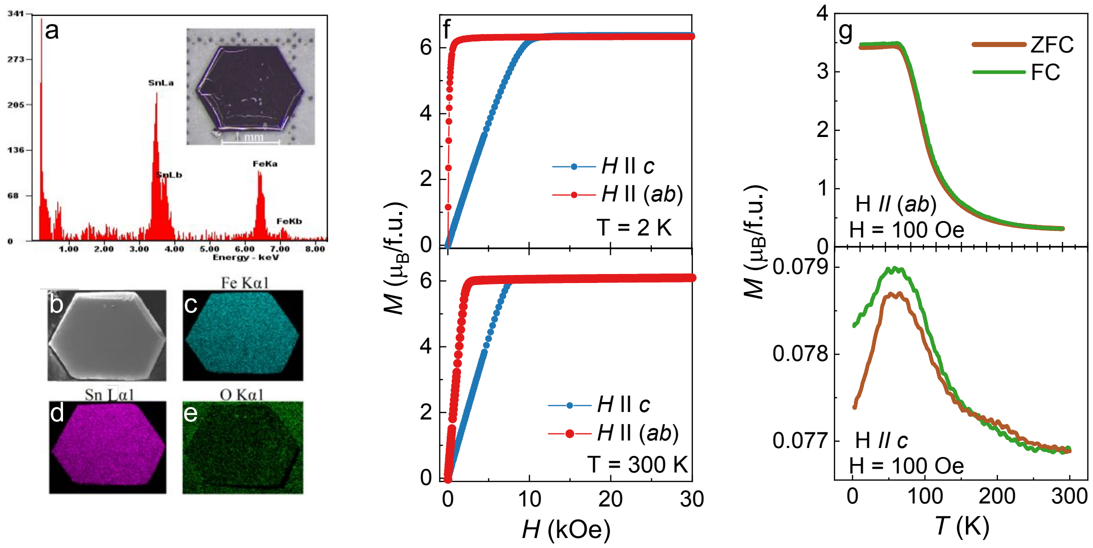

Figure S2. Energy dispersive X-ray spectroscopy (EDS) spectra (a), scanning electron microscopy (SEM) image (b), and elemental mapping (c-e) and of the single crystalline Fe_3Sn_2. The inset in a show an optical image of the selected Fe_3Sn_2. (f) Magnetization curves as a function of the magnetic field measured at 2 K (upper panel) and 300 K (lower panel), with the magnetic field applied within the *ab* plane and along the *c* axis, respectively. (b) Temperature dependence of zero-field-cooled (ZFC) and field-cooled (FC) magnetization measured at 100 Oe applied in the *ab* plane (upper panel) and along the *c* axis (lower panel).

The chemical composition of selected 1x1 mm^2 Fe_3Sn_2 crystal was verified using energy-dispersive X-ray spectroscopy (EDS) with a ZEISS Crossbeam 550. The scattered data were collected $5x10^6$ times from areas of 50-200 $μm^2$ at different regions of the sample. The EDS spectra in Figure S2a indicates the pure Fe-Sn phase. The averaged chemical composition was determined to be $Fe_{3.02(1)}Sn_{1.98(1)}$ ($Fe_{60.41}Sn_{39.56}$ in at.%), confirming the Fe_3Sn_2 stoichiometry within the accuracy limits of the EDS technique.

The SEM image highlights the high-quality surface of the as-grown Fe_3Sn_2 crystal, while the elemental mapping reveals a homogeneous dispersion of Fe (blue) and Sn (pink) atoms across the sample surface (Figure S2b-d). The small oxygen intensity detected on the surface of Fe_3Sn_2 is attributed to the scattering from the double-sided carbon tape used to mount the sample.

The magnetic susceptibility and magnetization of the selected sample were investigated by Quantum Design MPMS3 in the temperature range of 2-300 K and magnetic fields up to 3 T. Figures S2f shows the magnetization curves of Fe_3Sn_2 measured for magnetic fields applied within the *ab* plane and along the *c* axis. At 2 K, full saturation of magnetization M_s along the *a* axis is achieved in fields ~1kOe, while along *c* axis about 10 kOe are needed to reach full saturation. The calculated M_s ~2.09 μB/Fe, which is in good agreement with previous results reported for the same conditions [Wang, et al., Anomalous Hall effect in a ferromagnetic Fe3Sn2 single crystal with a geometrically frustrated Fe bilayer kagome lattice, Phys. Rev. B 94, 075135 (2016), Kumar et al., Magnetotransport as a diagnostic of spin reorientation: Kagome ferromagnet as a case study, Phys. Rev. B 100, 214420 (2019)]. With increasing temperature, the saturation field increases for *H*||*a* and decreases for *H*||*c*.

Figure S2g represents the zero-field-cooled (ZFC) and field-cooled (FC) temperature-dependence of magnetization measured at 100~Oe for two configurations. The resulting magnetization shows a continuous increase as the temperature decreases to 60 K. Below this temperature, the magnetization within the *ab* plane saturates, while it decreases for *H*||*c*. The latter is attributed to the spin reorientation effect [30, Kumar et al., Magnetotransport as a diagnostic of spin reorientation: Kagome ferromagnet as a case study, Phys. Rev. B 100, 214420 (2019)]. The ZFC-FC magnetization exhibits a minor hysteresis, likely due to the dynamics of the magnetic microstructures.

3. Field-controlled magnetic states in Fe_3Sn_2

Figure S3 shows a bright-field TEM image (Fig. S3a) and Fresnel defocus images (Fig. S3b-d) of different magnetic states in the Fe_3Sn_2 lamella, depending on the direction of the saturating magnetic field. Strain was not applied to generate these states. The magnetic field was applied using the conventional microscope objective lens. Each image was recorded at magnetic remanence. Figure S3b shows dipolar skyrmions after a saturating field was applied in the out-of-plane direction, *i.e.*, parallel to the c-axis of Fe_3Sn_2. Figures S3c, d show stripe domains aligned with the marked vertical and horizontal in-plane components of the saturating field, which was applied by tilting the specimen.

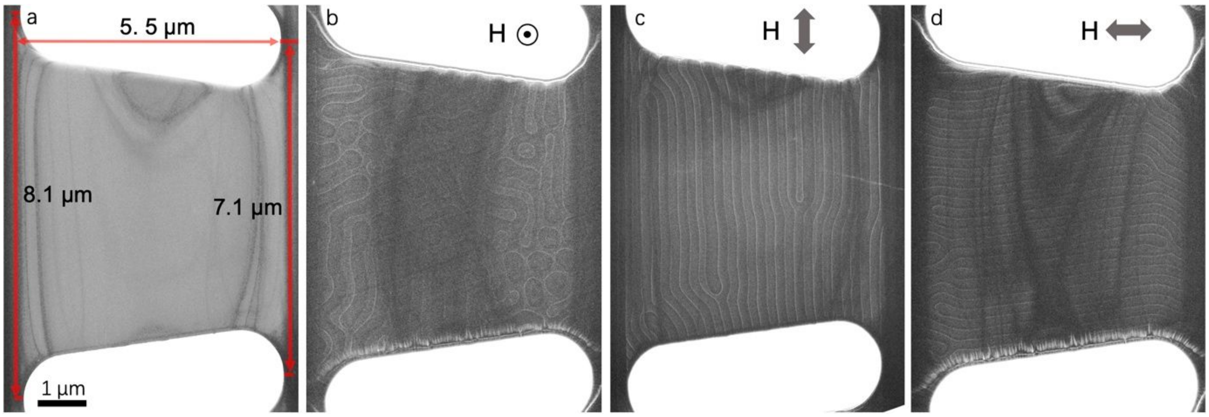

Figure S3. (a) Bright-field TEM image of Fe_3Sn_2 recorded close to focus. (b) Fresnel defocus image of dipolar skyrmions after the application of a saturating out-of-plane field. (c, d) Fresnel defocus images of stripe domains after the application of saturating fields, whose in-plane directions are marked. The defocus values are 0.6 mm in (b-d).

4. Helicities of dipolar skyrmions

The helicities of dipolar skyrmions were defined using Fresnel defocus images and electron optical phase images extracted from off-axis electron holography.

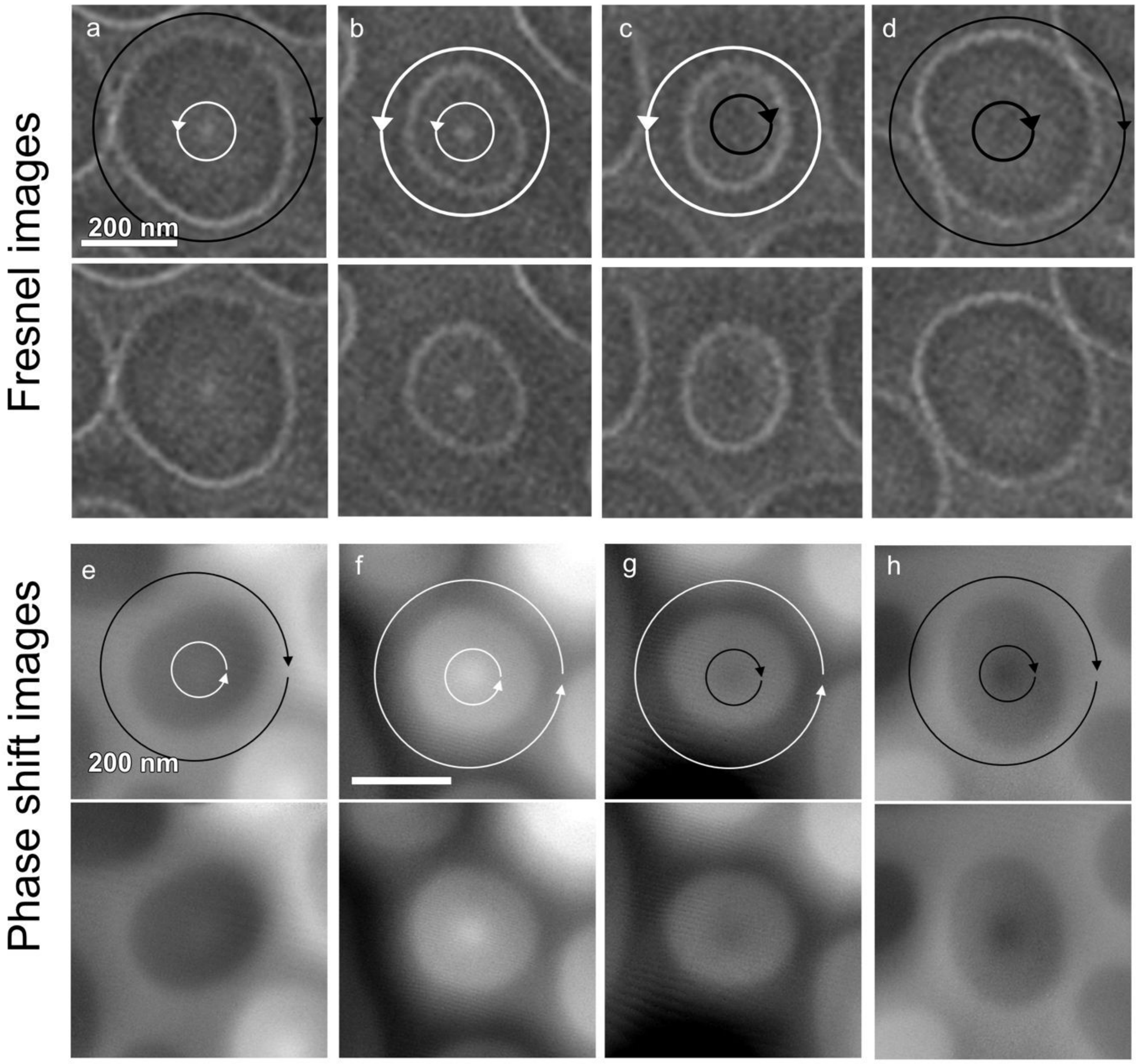

Figure S4. (a-d) Fresnel defocus images and (e-h) phase shift images of the dipolar skyrmions (type I structures) presented with the clockwise (black arrowhead) and counterclockwise (white arrowhead) rotation marks and without it.

5. Schematic diagrams of magnetic structures and Fresnel defocus images

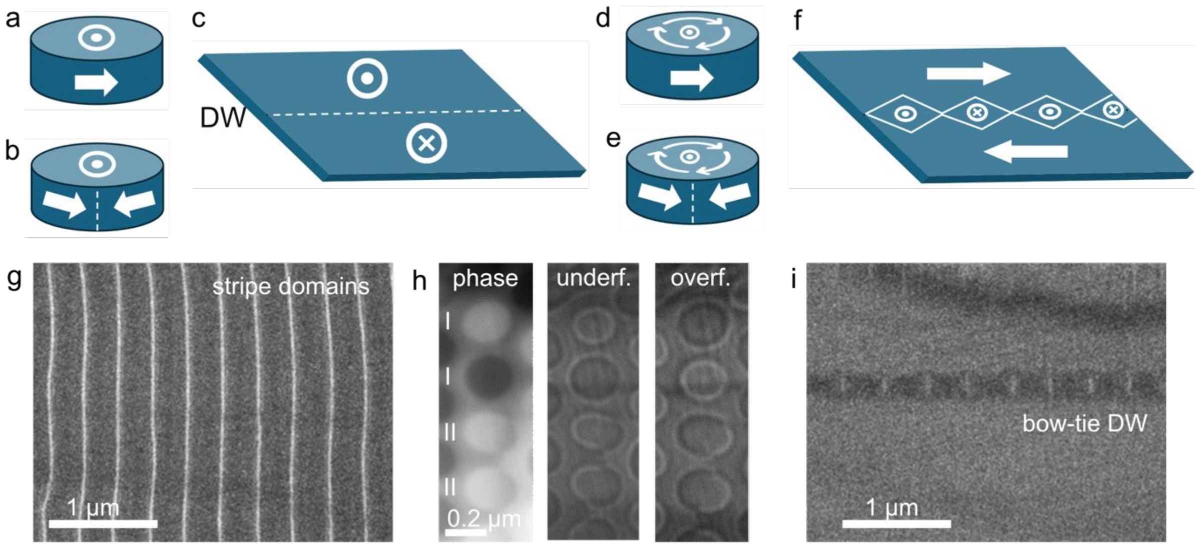

Figure S5. Schematic diagrams of magnetic (a) bubble type-I, (b) type-II, (c) stripe domains with out-of-plane magnetization, (d) dipolar skyrmion type-I, (e) dipolar skyrmion type-II, and (f) bow-tie domain wall between two in-plane magnetic domains. The dashed lines in (b, c, e) mark a domain wall (DW). (g) Fresnel defocus image of stripe domains. (h) Phase shift image and corresponding Fresnel defocus images of four dipolar skyrmions with type-I and type-II structures. The underfocus and overfocus values are 0.4 mm. (i) Fresnel defocus image of a bow-tie domain wall. The defocus values in (g, i) are 0.8 mm.

6. Fracture test

Figure S6 shows images of an Fe_3Sn_2 lamella containing regions of varying thickness. In the thinnest (60 nm) region, the magnetic state is markedly different from that in thicker (>100 nm) regions. In the thinnest region, the magnetization is aligned predominantly in the in-plane direction, both before and during straining. In this case, shape anisotropy is stronger than magnetocrystalline anisotropy. In Fig. S6c, in-plane magnetic domain formation occurs at lower strain in the 120-nm-thick region than in the 160-nm-thick region, confirming the importance of magnetocrystalline anisotropy, which is weaker in the thinner region. The lamella was further strained till it fractured, as shown in Fig. S6d. At this condition, the strain is relaxed in the two broken parts. The red arrow in Fig. S6d marks a dipolar skyrmion formed in the relaxed sample.

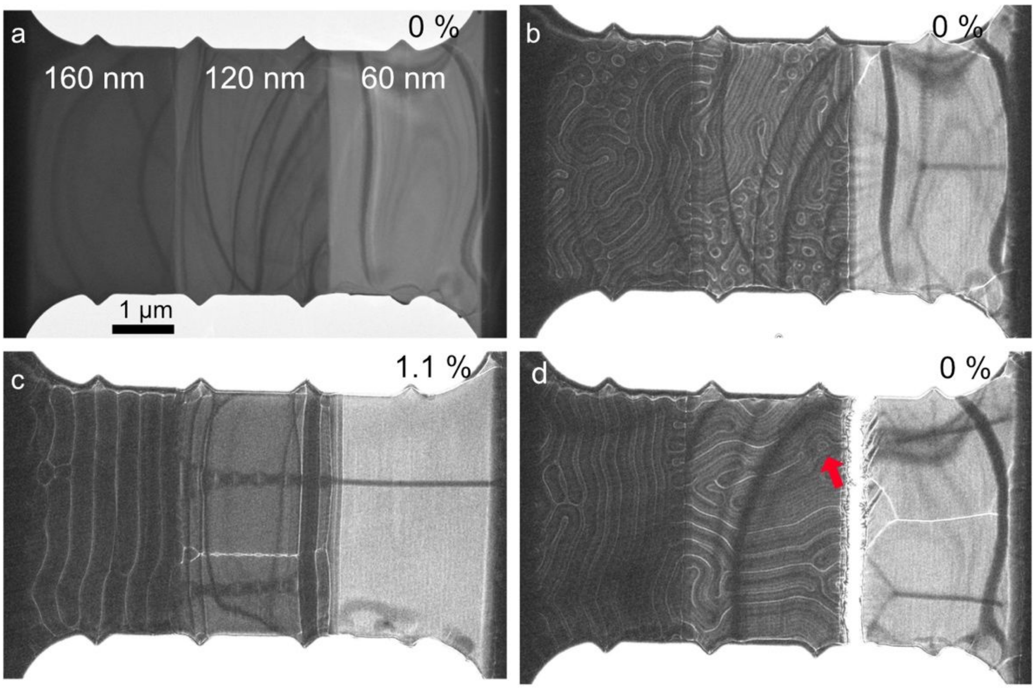

Figure S6. Formation of dipolar skyrmions in samples that had been highly strained through the fracture point. (a) Bright-field TEM image of a lamella of Fe_3Sn_2 containing regions of thickness 160, 120 and 60 nm. (b-d) Fresnel defocus images recorded during stretching of the lamella until it fractured. A dipolar skyrmion generated after the lamella had fractured is marked by a red arrow in (d). The strain levels are indicated in the upper right corners. The images were recorded in magnetic-field-free conditions. The defocus values in (b-d) are 1.6 mm.

7. Merging of dipolar skyrmions of opposite helicities

Figure S7 shows the merging of two dipolar skyrmions of opposite helicity in the external region of an Fe_3Sn_2 lamella, resulting in parallel field alignment at their peripheries. The central regions have the same helicity. Upon merging, the core is not visible and a sizeable in-plane magnetic induction is observed.

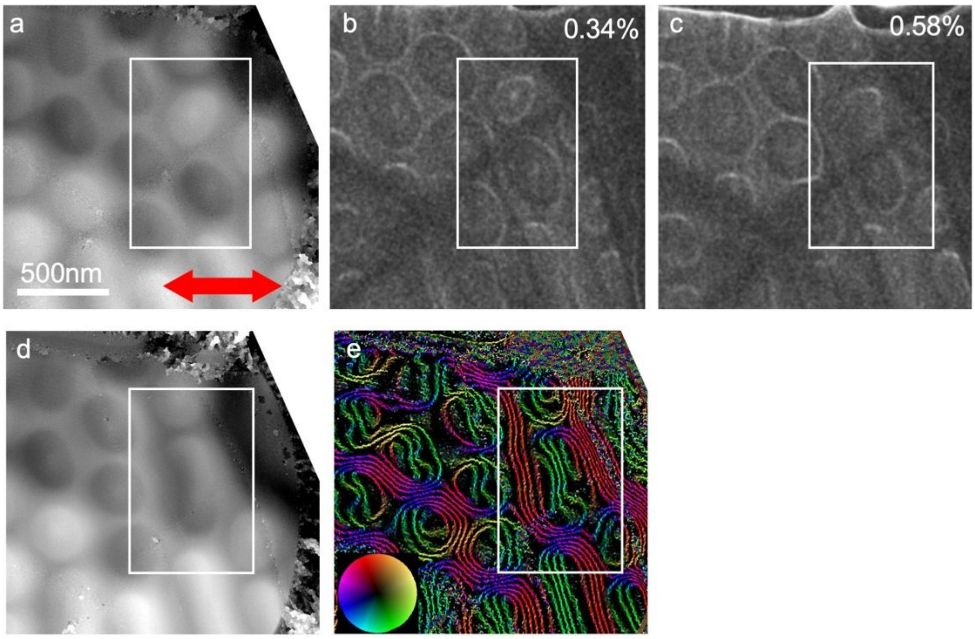

Figure S7. (a) Electron optical phase shift image of dipolar skyrmions reconstructed using off-axis electron holography. The red arrow marks the strain direction. (b, c) Fresnel defocus images recorded at different strain levels. (d, e) Electron optical phase shift image and corresponding magnetic induction map of the merged particles. The experiments were carried out with the sample in magnetic-field-free conditions. The defocus values in (b, c) are 0.7 mm. The phase contour spacing in (e) is 2π/3 radians. The frame marks approximately the same region in each panel.

Description of supplementary movies S1 and S2

S1. Strain-release cycle. Fresnel defocus images recorded during a strain-release cycle at magnetic remanence. The strain was applied in the horizontal direction. The strain values given in Fig. 2 were estimated after the experiment. The scale bar is 1 μm. The defocus value is 0.8 mm.

S2. Control of positions of magnetic skyrmions by stress. A 240 mT magnetic field was applied in the viewing direction to stabilize dipolar skyrmions. Stress was applied in the horizontal direction. By applying and removing stress, dipolar skyrmions could be moved directionally to the left and returned to their original positions. The scale bar is 1 μm. The defocus value is 1.2 mm.
